# A Novel Mechanism for Protein Delivery by the Type 3 Secretion System for Extracellularly Secreted Proteins

**DOI:** 10.1128/mBio.00184-17

**Published:** 2017-03-28

**Authors:** Farid Tejeda-Dominguez, Jazmin Huerta-Cantillo, Lucia Chavez-Dueñas, Fernando Navarro-Garcia

**Affiliations:** Department of Cell Biology, Centro de Investigación y de Estudios Avanzados del IPN (CINVESTAV-IPN), Mexico City, Mexico; UT Southwestern Med Center Dallas

**Keywords:** EspA filament, EspB-EspD pore, EspC, protein binding, protein translocation, secretion systems, enteropathogenic *E. coli*, autotransporter proteins, translocator proteins, YopH

## Abstract

The type 3 secretion system (T3SS) is essential for bacterial virulence through delivering effector proteins directly into the host cytosol. Here, we identified an alternative delivery mechanism of virulence factors mediated by the T3SS, which consists of the association of extracellularly secreted proteins from bacteria with the T3SS to gain access to the host cytosol. Both EspC, a protein secreted as an enteropathogenic *Escherichia coli* (EPEC) autotransporter, and YopH, a protein detected on the surface of *Yersinia*, require a functional T3SS for host cell internalization; here we provide biophysical and molecular evidence to support the concept of the EspC translocation mechanism, which requires (i) an interaction between EspA and an EspC middle segment, (ii) an EspC translocation motif (21 residues that are shared with the YopH translocation motif), (iii) increases in the association and dissociation rates of EspC mediated by EspA interacting with EspD, and (iv) an interaction of EspC with the EspD/EspB translocon pore. Interestingly, this novel mechanism does not exclude the injection model (i.e., EspF) operating through the T3SS conduit; therefore, T3SS can be functioning as an internal conduit or as an external railway, which can be used to reach the translocator pore, and this mechanism appears to be conserved among different T3SS-dependent pathogens.

## IMPORTANCE

The type 3 secretion system is essential for injection of virulence factors, which are delivered directly into the cytosol of the host cells for usurping and subverting host processes. Recent studies have shown that these effectors proteins indeed travel inside an “injectisome” conduit through a single step of translocation by connecting the bacterium and host cell cytoplasms. However, all findings are not compatible with this model. For example, both YopH, a protein detected on the surface of *Yersinia*, and EspC, an autotransporter protein secreted by enteropathogenic *E. coli*, require a functional T3SS for host cell translocation. Both proteins have an intermediate extracellular step before their T3SS-dependent translocation. Here, we show an alternative delivery mechanism for these extracellularly secreted virulence factors that are then incorporated into the T3SS to enter the cells; this novel mechanism coexists with but diverges from the canonical injection model that involves the passage of the protein inside the injectisome.

## INTRODUCTION

The type 3 secretion system (T3SS) is a highly conserved multimolecular protein assembly which is common in many enteropathogenic bacteria. This system is essential for virulence and enables productive interaction between the bacteria and the eukaryotic target cell by delivering directly into the cytosol of the host cells effector proteins that usurp and subvert host processes (1, 2). Great progress has been made in the last decade toward defining the architecture of T3SSs using prototypic bacteria, including *Yersinia* spp., *Shigella flexneri*, *Salmonella enterica*, enteropathogenic *Escherichia coli* (EPEC), and enterohemorrhagic *E. coli* (EHEC) (3–5). T3SSs contain supramolecular structures known as needle complexes (6). Cryo-electron microscopy and X-ray crystallography studies of isolated T3SS core complexes and their components have shown that its structure includes membrane-embedded oligomeric rings that are connected by a transperiplasmic rod to a hollow needle through which unfolded effectors are secreted (7–9). It has been shown that this syringe-like, membrane-embedded “injectisome” functions as a conduit, which was visualized using heterologous substrates that become trapped within the secretion path *in situ* inside the injectisome conduit while in action (10).

Three proteins (one hydrophilic protein and two hydrophobic proteins) known as translocators are themselves secreted via the T3SS and are required for the transit across the host cell membrane (11). However, the degree to which they are conserved is quite variable in all T3SS proteins, including such translocator proteins. In EPEC and EHEC strains, the hydrophilic translocon component *E*PEC *s*ecreted *p*rotein *A* (EspA) is related to LcrV from *Yersinia* spp. or IpaD from *S. flexneri* only distantly, but all of them share the coiled-coil structure (12). EspA binds to the needle protein EscF but does not form a pentameric ring at the needle tip such as LcrV does (13). Instead, EspA apparently tethers the bacterium to the host cell by forming a sheath-like filament extending about 93 nm on average (14, 15). The EspA filament is a helical tube with 5.6 subunits per turn, an outer diameter of 12 nm, and an inner diameter of 25 Å (16). On the other hand, the other two hydrophobic translocators have predicted transmembrane helices. *Yersinia* YopD (EspB in EPEC and EHEC) and YopB (EspD) can be considered prototypes of the hydrophobic translocon components thought to form a pore in the host cell membrane through which effector proteins pass (17). In the case of EPEC, the T3SS is located in a 35.6-kb pathogenicity island termed LEE (locus of enterocyte effacement) (18). LEE is organized into five polycistronic operons: LEE1, LEE2, and LEE3 encode the T3SS or injectisome; the products encoded by LEE4 comprise the T3SS-secreted translocator proteins EspA, EspB, and EspD. Through this injectisome, a LEE5 effector, Tir, is injected directly into the cell and is inserted into the membrane, exposing an extracellular domain that is recognized by intimin (an EPEC membrane adhesin), also encoded by LEE5 (19). Intimin-Tir interactions lead to elicitation of a histopathologic lesion formed at the mucosal intestinal surface that displays a pedestal-like structure, known as an attaching and effacing (AE) lesion (20). Other LEE-encoded effector proteins (EspG, EspZ, EspH, Map, and EspF) are also injected into the cell during infection (20). Notably, the EPEC T3SS also translocates non-LEE-encoded effectors, including NleA/EspI, EspJ, EspL, EspO, NleB, NleC, NleD, NleE, NleF, NleG, NleH, and Cif (21). All these effectors hamper different aspects of the cell physiology, including subverting innate immune pathways, specifically those involved in phagocytosis, host cell survival, apoptotic cell death, and inflammatory signaling, which are all required to cause disease (20, 22).

A second pathogenicity island of EPEC that encodes EspC has been identified in pathogenic EPEC1 strains. Unlike proteins secreted by the T3SS, EspC secretion is mediated by the type V secretion system (T5SS) or autotransporter system (23, 24). A recent study showed that *espC* is one of the most prevalent genes among those encoding autotransporter proteins in both typical and atypical EPEC strains (25). EspC is able to exert cytotoxic effects on epithelial cells, and these effects depend on its serine protease motif (26). EspC protein has to get inside the cells in order to cleave intracellular targets such as fodrin and focal adhesion proteins such as focal adhesion kinase (FAK) and paxillin (27) as well as proteins related to the apoptosis cascade such as pro-caspase 3 (28). The cleavage of these intracellular targets by EspC leads to cell rounding and detachment followed by cell death through apoptosis and necrosis (28). Interestingly, EspC is not efficiently internalized under nonphysiological conditions (as a purified protein), because no receptor is involved in its uptake. However, EspC physiologically secreted by EPEC is efficiently internalized during the interaction of EPEC with epithelial cells (29). Thus, a key step for the cell damage induced by EspC is its internalization process; EspC is the first protein found in tissue culture medium containing infected cells and is found long before the T3SS proteins (30). EspC translocation depends on EPEC and host cell contact, and EspC secretion is stimulated by EPEC in the presence of cell culture medium (as T3SS effectors of EPEC) and enhanced by the presence of epithelial cells (29), which correlates with the EspC regulation by Ler, even though EspC is not located in LEE such as are the classical T3SS effector proteins that are also regulated by Ler (31). Furthermore, after EspC secretion by the T5SS, and once in the extracellular medium, EspC is efficiently translocated into the epithelial cells in a T3SS-dependent manner (32). These data suggest that EspC translocation inside the epithelial cells appears to involve a singular translocation mechanism. Here, we provide evidence supporting the concept of the involvement of a novel mechanism to explain the translocation of proteins inside the host cell by the T3SS for proteins that have already been secreted into the extracellular medium, which diverges from the canonical microinjection model that involves the passage of the protein inside the injectisome that connects the bacterial cytoplasm to the host cell cytoplasm.

## RESULTS

### EspC interacts strongly with EspA mainly through its Middle segment.

**FIG 1:**
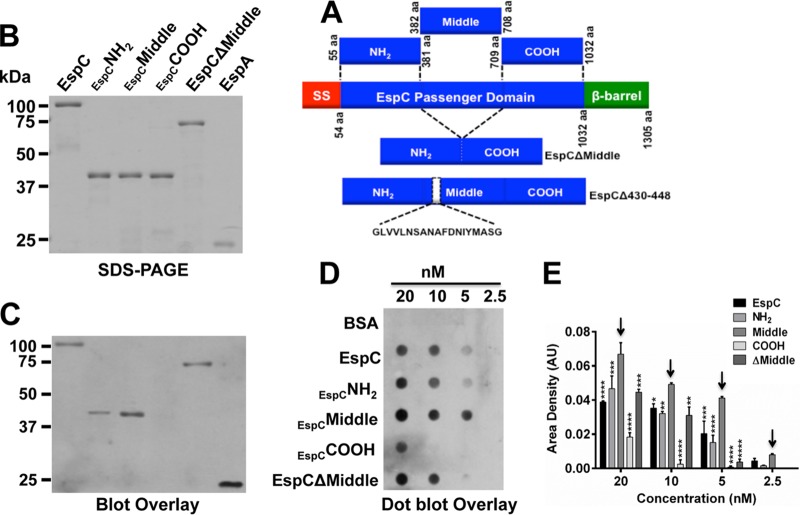
EspA interacts mainly with the EspC Middle segment. (A) Schematic representation of EspC protein domains and proteins generated by cloning EspC segments or deleting specific sequences. The EspC protein is an autotransporter protein and comprises a signal sequence (SS; aa 1 to 53), a passenger domain (PD; aa 54 to 1018) containing the serine protease domain (S256), and the translocation unit (β-barrel; aa 1018 to 1305). The DNA encoding the passenger domain was divided into three 0.97-kb fragments to be cloned in pRSET-A for obtaining 37-kDa N-terminal (NH_2_; aa 55 to 381), middle (middle; aa 382 to 708), and C-terminal (COOH; aa 709 to 1032) protein segments. Additionally, two truncations were generated by deletion of the Middle segment (EspCΔMiddle) and residues 430 to 448 (His-EspCΔ430-448). All constructs contained a 6×His tag in their N-terminal side, which was used for recombinant protein purification. (B) SDS-PAGE of purified EspC and its derivate proteins. EspC, EspC His-NH_2_, His-Middle and His-COOH segments, His-EspCΔMiddle, and His-EspA were run in a 12% gel. (C) Blot overlay for EspC-EspA interaction. The proteins described for panel A were run in an SDS-PAGE and transferred to a PVDF membrane. The membrane was probed with His-EspA protein, and its interaction with other proteins was detected by Western blotting using anti-EspA antibodies. The image is representative of results from four independent experiments. (D) Dot blot overlay for interaction of nondenatured EspC and its derivatives with His-EspA. The proteins described for panel A were dropped on a nitrocellulose membrane using double serial dilutions from 20 nM to 2.5 nM of EspC, EspC His-NH_2_, His-Middle or His-COOH segments, His-EspCΔMiddle, or BSA (as a negative control). The membrane was incubated with His-EspA, and its interaction with these proteins in the membrane was detected by Western blotting using anti-EspA antibodies. (E) Densitometry analyses of data from three independent experiments performed as described for panel C, showing data from a representative one. Data (means ± standard errors of the means [SEM]) represent results of comparisons of levels of His-EspA binding to the different proteins in the nitrocellulose membrane. Statistical analysis was performed using two-way analysis of variance (ANOVA) followed by Dunnett’s multiple-comparison test for comparisons to the Middle segment (*, *P* < 0.05; **, *P* < 0.01; ***, *P* < 0.001; ****, *P* < 0.0001). Arrows show the highest level of interaction of His-EspA with the EspC His-Middle segment. AU, arbitrary units.

In order to find the EspC segment interacting with EspA, we began by simply dividing the passenger domain into three segments. Thus, the sequence encoding the passenger domain was divided into three 0.97-kb fragments (Fig. 1A), the N-terminal fragment (NH_2_: amino acids [aa] 55 to 381), the Middle fragment (aa 382 to 708), and the C-terminal fragment (COOH; aa 709 to 1032), which were cloned in pRSET-A. Histidine-tagged segment proteins (42 kDa) and His-EspA were purified in nickel-nitrilotriacetic acid (NTA) columns, while the whole EspC passenger domain was purified from supernatants by filtration through 100-kDa-cutoff membranes (Fig. 1B). Interactions of these purified proteins with His-EspA were analyzed by blot overlay; proteins were run in SDS-PAGE, transferred to polyvinylidene difluoride (PVDF) membranes, and probed with His-EspA. Anti-EspA antibodies detected the interaction of EspA with native EspC but not with the corresponding COOH segment. The NH_2_ segment and the Middle segment were also able to interact with EspA, but the interaction was stronger with the Middle segment. The strongest interaction, i.e., the EspA-EspA interaction, was detected in the positive control (Fig. 1C), as expected since EspA subunit polymerizes to form the EspA filament (16). The sensitivity of the interaction between EspC segments and EspA was then assayed by performing a dot blot overlay under nondenaturing conditions using serial dilutions of EspC (20, 10, 5, and 2.5 nM) to reach a final dilution to analyze the interaction. Bovine serum albumin (BSA) was used as a negative control. The most sensitive EspA interaction was detected for the EspC Middle segment, which revealed a strong positive signal with anti-EspA with as low a concentration as 5 nM EspC Middle segment (Fig. 1D). The NH_2_ segment and the full-length EspC showed similar results for interactions with EspA, with sensitivities that were lower than those seen with the Middle segment, while the COOH segment interacted weakly with EspA and only at a high (20 nM) concentration (Fig. 1D). Moreover, densitometry of positive dots revealed that the interaction of the Middle segment with EspA was more pronounced than for the other segments or the full-length EspC at all concentrations tested (Fig. 1E).

**FIG 2:**
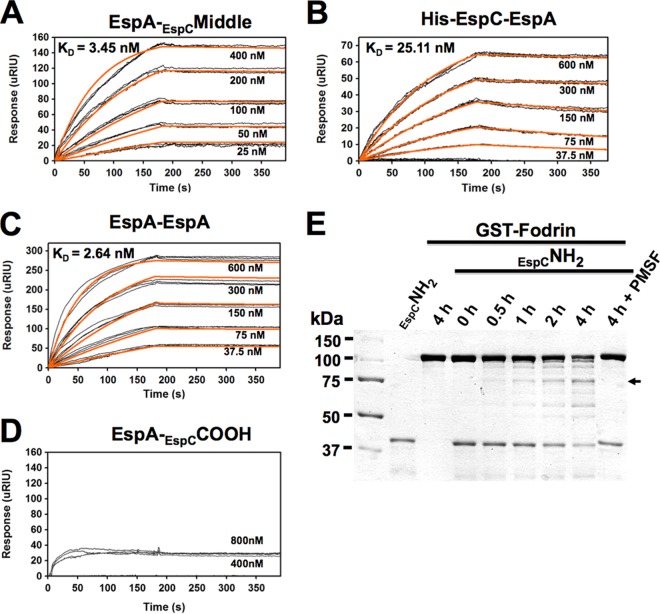
Function of the EspC Middle and NH_2_ segments. (A to C) Affinity of His-EspA for the EspC Middle segment (A), both His-EspC and His-EspA (B), and His-EspA itself (C). (D) As a negative control, the affinity of His-EspA for the EspC COOH segment was tested. Increasing concentrations of His-EspA or the EspC His-Middle segment were perfused over an SPR sensor coated with His-EspA, or increasing concentrations of His-EspA were perfused over an SPR sensor coated with His-EspC. Responses (μRIU) observed for each protein concentration are shown and were used to calculate the value for affinity (*K*_*D*_) between EspA and EspA or the EspC Middle segment or the whole EspC protein. (E) Fodrin degradation by EspC His-NH_2_ segment. Purified GST-fodrin and EspC His-NH_2_ segment proteins (2 μg) were incubated for various times. The EspC His-NH_2_ segment and GST-fodrin were also separately incubated for the maximum time (4 h), and GST-fodrin was incubated for 4 h with the EspC His-NH_2_ segment pretreated with the serine protease inhibitor PMSF, as negative controls. After the interaction, the proteins were separated by SDS-PAGE. The arrow indicates 75-kDa fodrin degradation subproducts.

In order to test whether the Middle segment was required as part of the full-length EspC context for the strong interaction with EspA, we designed an EspCΔMiddle construct, which contained the passenger domain but lacked the Middle segment, inserted into the pRSET-A vector (Fig. 1B). Interestingly, EspA was able to bind EspCΔMiddle and the NH_2_ segment at similar magnitudes in both blot overlay assays and dot blot overlay assays (Fig. 1C and D), as expected since the middle-segment mutant still contains the sequence of the NH_2_ segment. However, EspA binding to EspCΔMiddle was lower than that seen between EspA and the Middle segment. These data suggest that both the EspC N-terminal segments and Middle segments participate in binding EspA, with the contribution of the Middle segment being more important. To further understand this interaction, we calculated the equilibrium dissociation constant (*K*_*D*_) between EspA and the Middle segment of EspC. To do that, increasing concentrations of this Middle segment (25 nM to 400 nM) were perfused over a surface plasmon resonance (SPR) sensor coated with EspA. Perfusion of EspC Middle segment over the sensor slide generated an increase in the refractive index unit (RIU) value, thus indicating direct binding of the Middle segment to EspA (Fig. 2A). On rates, off rates, and equilibrium dissociation constants (*K*_*D*_) calculated from the aggregate data using the different Middle segment concentrations are shown in Fig. 2A. EspA bound to the EspC Middle segment with a *K*_*D*_ of 3.45 ± 0.96 nM. Following the same approach, the *K*_*D*_ of the interaction between EspA and the whole EspC protein was 25.11 ± 2.4 nM (Fig. 2B), which was an order of magnitude greater than that of the interaction between the EspC Middle segment and EspA. Given that the *K*_*D*_ of the EspA-EspA interaction has not been previously reported, we also analyzed this by SPR analysis as a positive control. Increasing concentrations (37.5 nM to 600 nM) of His-EspA were perfused over an SPR sensor coated with His-EspA, which generated an increase in the RIU, allowing determination of a *K*_*D*_ value of 2.64 nM (Fig. 2C). On the other hand, consistent with previous results determined in blot and dot blot overlay experiments, EspA and the COOH segment of EspC did not generate an increase in the refractive index unit (RIU), thus indicating the lack of binding of the COOH segment to EspA (Fig. 2D).

Since the NH_2_ segment had the ability to interact with EspA and also contains the serine protease motif in its sequence, we tested if this NH_2_ segment is able to retain the proteolytic activity. Thus, the EspC NH_2_ segment was incubated with glutathione *S*-transferase (GST)-fodrin (fodrin is a target protein of EspC) for increasing time intervals (0, 0.5, 1, 2, and 4 h). The EspC NH_2_ segment was able to cleave fodrin in a time-dependent manner to generate increasing amounts of a 75-kDa degradation subproduct, correlating with a decrease in the GST-fodrin protein band (Fig. 2E). In contrast, preincubation of the EspC NH_2_ segment with phenylmethylsulfonyl fluoride (PMSF) (a serine protease inhibitor) completely abolished this proteolytic activity even at 4 h of interaction. These data indicate that the EspC NH_2_ segments of 37 kDa retained the catalytic activity of the full-length protein, suggesting that this segments helps the interaction with EspA and, once inside the cells, uses its catalytic site to cleave intracellular targets.

### The middle segment is required for EspC translocation into the epithelial cells.

**FIG 3:**
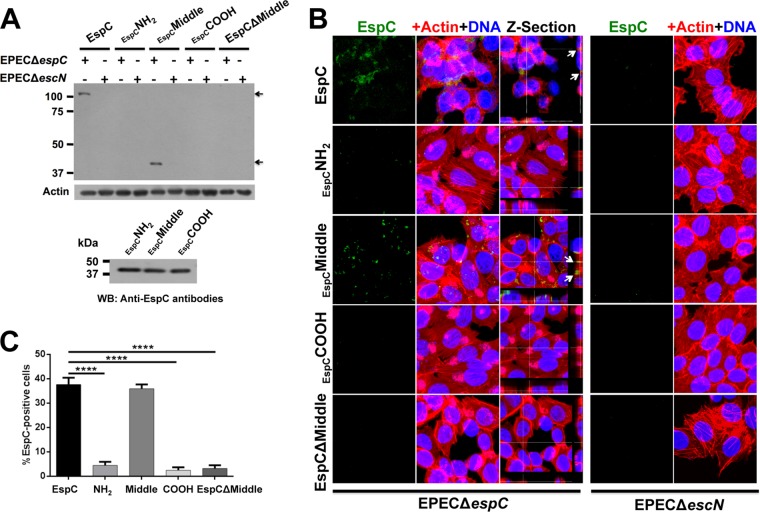
Only full-length EspC and EspC Middle segment are translocated inside the cells using the T3SS. (A) Detection of EspC in cytoplasmic fraction. Epithelial cells were infected with either EPEC Δ*espC* or EPEC Δ*escN* and coincubated with EspC, EspC His-NH_2_, His-Middle or His-COOH segments, or His-EspCΔMiddle mutant proteins (0.9 μM) for 4 h. Infected cells were fractionated, the cytoplasmic fraction was analyzed by Western blotting (WB) using polyclonal anti-EspC antibodies, and actin detection was used as a loading control. The image is representative of results from four independent experiments. Arrows indicate EspC detected in the cytoplasmic fraction. The bottom panel shows similar levels of detection of the purified EspC segments by the polyclonal anti-EspC antibodies by Western blotting. (B) Internalization of exogenous EspC and EspC Middle segment into epithelial cells during infection by EPEC Δ*espC*. Epithelial cells were infected with either EPEC Δ*espC* or EPEC Δ*escN* and coincubated with EspC, EspC His-NH_2_, His-Middle or His-COOH segments, or His-EspCΔMiddle proteins (0.9 μM) for 3 h. Infected cells were fixed, permeabilized, and stained for confocal microscopy. EspC was detected by using anti-EspC antibodies followed by anti-mouse antibodies conjugated to fluorescein isothiocyanate (FITC; green), and the actin cytoskeleton was detected with rhodamine-phalloidin (red) and DNA with TO-PRO-3 iodide (blue). EspC detection results are individually shown in the green channel as well as the maximal projections of the three channels (merged panels) and a middle *z*-section of the three channels. Arrows show EspC detection inside the cells in the *z*-sections. (C) Quantification of EspC-positive cells (green cells). Three experiments whose results were similar to those shown in panel B were analyzed by counting the number of EspC-positive cells. Data from nine images of each condition are expressed in percentages (means ± SEM). Statistical analysis was performed using one-way ANOVA and Holm-Sidak’s test (***, *P* < 0.0001).

In order to investigate which EspC segment is needed for EspC translocation through the T3SS, we combined the ability of EPEC Δ*espC* for translocating exogenous EspC (purified protein) and the use of the three EspC segments (NH_2_, Middle, and COOH). HEp-2 cells were infected with EPEC Δ*espC* or EPEC Δ*escN* (a mutant deficient for the T3SS formation) and coincubated with the same concentration (0.9 μM) of purified EspC or each of the EspC segments. The cytoplasmic fractions of these infected cells were analyzed by Western blotting using polyclonal anti-EspC antibodies, which recognize the three EspC segments (Fig. 3A). Exogenous whole EspC protein was efficiently translocated into cells infected with EPEC Δ*espC* but not into those infected with EPEC Δ*escN*, as previously reported (32). Similarly, none of the three EspC segments were translocated into cells infected with EPEC Δ*escN*, whereas only the EspC Middle segment was detected in the cytoplasm of EPEC Δ*espC*-infected cells; a typical result representative of four experiments is shown in Fig. 3A. These data indicate that a sequence in the EspC Middle segment, but not in the NH_2_ or COOH fragment, is required for EspC translocation into the epithelial cells. To corroborate these results, HEp-2 cells were infected with EPEC Δ*espC* or EPEC Δ*escN* and coincubated with EspC truncated in the Middle segment but containing the NH_2_ and COOH segments (EspCΔMiddle) and none of these bacterial mutants were able to translocate EspCΔMiddle inside the cells (Fig. 3A), strongly suggesting that the NH_2_ segment is needed for a partial EspC-EspA interaction but not for EspC translocation, while, coincidentally, the EspC Middle segment is needed for a strong EspC-EspA interaction and for EspC translocation.

To further confirm that the EspC Middle segment is needed for EspC translocation, we used our exogenous protein translocation model. HEp-2 cells were infected with either EPEC Δ*espC* or EPEC Δ*escN*, and we performed coincubations with the different purified proteins: whole EspC, EspC NH_2_, Middle, COOH, and EspCΔMiddle. Infected cells were prepared for confocal microscopy by staining the actin cytoskeleton with rhodamine-phalloidin (red) and the cell nuclei with TO-PRO-3 (blue) and by EspC translocation using anti-EspC antibodies followed by a secondary antibody labeled with fluorescein (green). As expected, in cells infected by EPEC Δ*espC*, the whole EspC protein was detected as green marks inside the cells and its internalization was confirmed by using *z*-section images, while this internalization was undetectable in cells infected with the T3SS functionally defective mutant (EPEC Δ*escN*) (Fig. 3B). In fact, EPEC Δ*escN* infection did not allow the translocation of any of the exogenous proteins tested. In sharp contrast, the EspC Middle segment was detected inside the cytoplasm of EPEC Δ*espC*-infected cells, but not EspC NH_2_, COOH, or EspCΔMiddle, and the translocation of the EspC Middle segment was confirmed by using *z*-section images (Fig. 3B). Quantification of those cells that were positive for EspC marks showed that, indeed, only the EspC Middle segment was able to get inside the cells, and these data were statistically supported (Fig. 3C). Together, these data indicate that EspC uses a sequence in its Middle segment for EspA binding and for translocating inside the infected cells through the T3SS.

### A YopH-shared sequence in the EspC Middle segment is required for its translocation.

**FIG 4:**
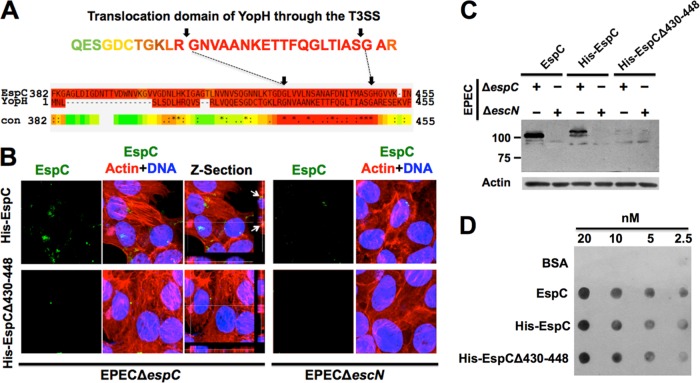
A translocation motif inside the EspC Middle segment with homology to YopH is required for EspC translocation by the T3SS. (A) Homology of EspC and YopH in the internalization domain for the T3SS. Amino acid sequences of YopH (P08538) and EspC (Q9EZE7) were aligned using PSI-Coffee. A scale consisting of values from 0 (blue, poorly supported) to 9 (dark red, strongly supported) was used; scores higher than 5 (i.e., yellow/orange/red) indicate sequences that were quite likely to be correctly aligned. A 19-aa region shared between EspC and the internalization domain of YopH (31 aa) through the T3SS is shown. (B) The 19-aa region of EspC shared with YopH is required for EspC internalization. Epithelial cells were infected with either EPEC Δ*espC* or EPEC Δ*escN* and coincubated with His-EspC or His-EspCΔ430-448 proteins (0.9 μM) for 3 h. Infected cells were fixed and stained for confocal microscopy. EspC was detected by using anti-EspC antibodies followed by anti-mouse antibodies conjugated to fluorescein isothiocyanate (FITC; green); the actin cytoskeleton was detected with rhodamine-phalloidin (red) and DNA with TO-PRO-3 iodide (blue). Panels representative of each condition from three independent experiments are shown. EspC detection is individually shown in the green channel as well as the maximal projections of the three channels (merged images) and a middle *z*-section of the three channels. Arrows show EspC detection inside the cells in the *z*-sections. (C and D) EspC translocation activity, but not EspA binding activity, was blocked by using the EspCΔ430-448 protein. (C) EspCΔ430-448 translocation. Epithelial cells were infected with either EPEC Δ*espC* or EPEC Δ*escN* and coincubated with EspC, His-EspC, or His-EspCΔ430-448 proteins (0.9 μM) for 4 h. Cytoplasmic fractions were analyzed by Western blotting using anti-EspC antibodies, and actin detection was used as a loading control. (D) Binding of His-EspCΔ430-448 to His-EspA. EspC, His-EspC, or His-EspCΔ430-448 proteins were dropped on a nitrocellulose membrane using double serial dilutions from 20 nM to 2.5 nM. BSA was used as a negative control. The membrane was incubated with EspA, and its interaction with these proteins in the membrane was detected by Western blotting using anti-EspA antibodies. Representative images from three independent experiments are shown in panels C and D.

In order to identify a functional motif inside the EspC Middle segment and considering that the EspA-EspB-EspD proteins interacted through coiled-coil domains, we initially looked for this type of motif inside the Middle segment of EspC. Coiled-coils prediction software could not detect such domains either for the Middle segment or for the EspC NH_2_ segment. Further analyses of EspC in FingerPRINTScan software (33) identified this protein as a serine protease of the IgA protease family (E value of 2.8 × 10^−11^), which was expected since EspC belongs to the SPATE (serine protease autotransporters of the *Enterobacteriaceae*) subfamily. Therefore, we decided to analyze only the sequence containing the EspC Middle segment (which has not the serine protease motif that is located in the NH_2_ segment) using the same software. The 37-kDa Middle segment protein provides a signature for the T3SS needle complex outer ring protein family with a certain fingerprint (E value of 8.9 × 10^3^). Interestingly, YopH, another bacterial protein that is translocated by externally added protein and that requires a functional T3SS (34), also had a certain fingerprint (E value of 1.3 × 10^3^) that provides a signature for the T3SS needle complex inner membrane protein family. Besides, the YopH high-scoring fingerprint (E value of 2.8 × 10^−42^) provides a signature for its known *Salmonella*/*Yersinia* type III secreted modular tyrosine phosphatases. Furthermore, analyses of the YopH and EspC Middle segments using PSI-Coffee software, which aligns distantly related proteins using homology extension (35), revealed high sequence homology of EspC with the translocation sequence of YopH (residues 18 to 49) (34), which consisted of 21 aa shared with EspC (residues 429 to 449) inside the Middle segment (Fig. 4A).

To determine if this shared sequence in the Middle segment is required for EspC translocation, a deletion of 19 aa of this region was engineered in the *espC* gene to produce the protein His-EspCΔ430-448 (Fig. 1A) and, for comparison purposes, the whole passenger domain of *espC* was also cloned into pRSET-A to obtain the protein His-EspC. HEp-2 cells were infected with EPEC Δ*espC* or EPEC Δ*escN* and coincubated with His-EspC or His-EspCΔ430-448, and protein translocation was analyzed by confocal microscopy. Infected cells were stained with rhodamine-phalloidin (F-actin) and TO-PRO-3 iodide (DNA), while EspC was detected with anti-EspC antibodies and a secondary antibody labeled with fluorescein. In strong support of our hypothesis, His-EspCΔ430-448 was unable to be translocated during the infection with either EPEC Δ*espC* or EPEC Δ*escN*, while the whole EspC passenger domain tagged with 6×His (His-EspC) was detected inside EPEC Δ*espC*-infected cells but not in cells infected with EPEC Δ*escN*; this translocation was confirmed by using *z*-section images (Fig. 4B). Quantification of EspC-positive cells (green) using nine images (as Fig. 4B) from three independent experiments showed that around 30% of EspC-positive cells were detected among those cells infected with EPEC Δ*espC* and coincubated with His-EspC but that only 4% were detected among those where His-EspCΔ430-448 was used instead of His-EspC. To further establish the requirement of this EspC translocation domain, the cytoplasmic fractions of cells treated as mentioned above were analyzed by Western blotting using anti-EspC antibodies. As expected, His-EspC and the native EspC were detected in the cytoplasm fractions of cells infected with EPEC Δ*espC* but not in those of cells infected with EPEC Δ*escN*, whereas His-EspCΔ430-448 was not detected in the cytoplasmic fraction of cells infected with either EPEC Δ*espC* or EPEC Δ*escN* (Fig. 4C); these data confirmed those obtained by confocal microscopy.

The failure of translocation of His-EspCΔ430-448 inside the cells may have been due to the loss of the EspA binding phenotype; in other words, this 19-aa sequence could be required for the EspC-EspA interaction. Therefore, to determine if this sequence is required for the EspC-EspA interaction, the interaction sensitivity of EspA with this purified His-EspCΔ430-448 protein was analyzed by dot overlay using serial dilutions (20, 10, 5, and 2.5 nM) and BSA was used as a negative control. The nitrocellulose membrane containing the different proteins was incubated with His-EspA. After the incubation, anti-EspA antibody was used to detect the interaction of EspA with His-EspCΔ430-448 and we found interaction sensitivity levels similar to those seen with native EspC or His-EspC (Fig. 4D). A quantitative analysis of three independent experiments showed no statistically significant differences among the results of all these treatments (data not shown). These data indicate that the EspC Middle segment contains a YopH-like EspC translocation sequence (residues 430 to 448) but that this sequence is not required for EspA binding, suggesting that the EspA binding motif and the translocation motif are separated in this 37-kDa Middle segment.

### EspC has a higher affinity for the EspA-EspD complex than for EspA alone.

**FIG 5:**
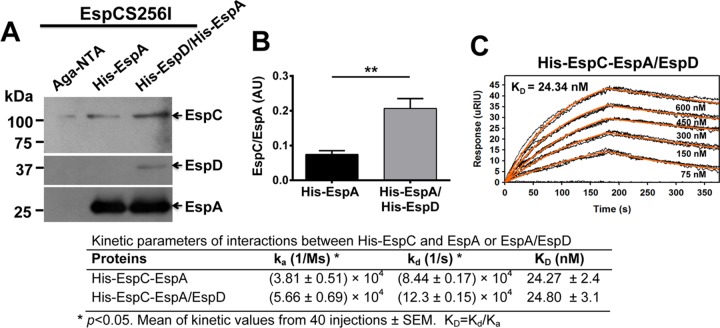
Interaction of EspA-EspC is increased in the presence of EspD. (A) His-EspA-EspC interaction in Ni-NTA columns. His-EspA and His-EspA/His-EspD were purified from BL21(p*espA*) and BL21(p*espA*) (p*espD*), respectively. Purified His-EspA or His-EspA/His-EspD (60 μg) was incubated with EspCS256I (20 μg) at 4°C for 2 h. The mixtures were passed through Ni-NTA columns, and the eluted fractions from the columns were analyzed by Western blotting using antibodies against EspC, EspA, and EspD. Aga, agarose. (B) Densitometry of His-EspA-EspCS256I interaction with and without His-EspD. The bands in the Western blot shown in panel A were quantified by densitometry. Data represent means ± standard errors of the results from four independent experiments. Treatments were compared by unpaired *t* test (**, *P* < 0.01). (C) Affinity of His-EspC for the His-EspA/His-EspD complex. Increasing concentrations of His-EspA/His-EspD proteins were perfused over an SPR sensor coated with His-EspC. Responses (μRIU) observed for each protein concentration are shown and were used to calculate the value for affinity (*K*_*D*_) between EspC and EspA/EspD; the kinetics parameters (*k*_*a*_ and *k*_*d*_ rates [*K*_*D*_]) of the interactions of EspC with EspA alone or with EspA/EspD are compared. Data represent means ± standard errors of the results from 40 different injections. Two-tailed unpaired *t* tests were performed (*P* values < 0.05).

Since EspC interacts with EspA using a binding motif, which is different from the EspC translocation sequence, we were expecting that, after its interaction with EspA, EspC must have been moving to the entry site by interacting with other components of the T3SS. We have previously shown that EspB is required for exogenous EspC translocation in EPEC Δ*espB*-infected cells but that there is not an interaction between EspC and EspB (32). Interestingly, it has been shown that EspA interacts with EspD but not with EspB (17). In fact, YopB homologues, including PopB, IpaB, and EspD, seem to play a primary role in pore formation (17). Therefore, we decided to search for any interaction of EspC with the connection between EspA and the EspD/EspB translocon to specifically test if the EspA-EspD complex interacts with EspC. To do this, first, we analyzed whether bacteria [BL21(p*espA*/p*espD*) cells] harboring *espA* and *espD* genes (both with a His tag) were able to form a heterologous protein complex. We found that this strain, coexpressing the two genes, was able to form a complex when the proteins were purified in a Ni-NTA agarose column and analyzed in nondenatured polyacrylamide gels using antibodies against EspA and EspD. Both antibodies were able to detect a big complex of around 100 to 150 kDa between these proteins under native conditions (data not shown). To analyze the interaction of EspC with the EspA-EspD complex versus EspA alone, EspCS256I (to avoid any proteolytic activity) was incubated for 2 h with either His-EspA [expressed in BL21 (p*espA*)] or His-EspA/His-EspD [coexpressed in BL21(p*espA*/p*espD*)], and then the mixtures were passed through a Ni-NTA agarose column and captured proteins were analyzed by Western blotting using anti-EspC, anti-EspD, or anti-EspA antibodies. EspC coeluted with His-EspA, but the amount of eluted EspC doubled when it coeluted with His-EspA/His-EspD (Fig. 5A), and this increase was statistically significant (Fig. 5B). SPR analyses of interactions between His-EspC and His-EspA/His-EspD were performed; increasing concentrations (75 nM to 600 nM) of His-EspA/His-EspD were perfused over an SPR sensor coated with His-EspC. The EspA/EspD complex caused a higher increase in the dissociation kinetics (*k*_*d*_ [1/s]) of EspC (Fig. 5C) than EspA alone did (see Fig. 2B). As a negative control, increasing concentrations (150 nM to 600 nM) of His–caspase-3 were perfused over an SPR sensor coated with His-EspC; no association between these proteins was detected (data not shown), and the results were similar to those seen for the interaction of EspA and the EspC COOH segment described above (see Fig. 2D). Thus, the EspA/EspD complex modified the kinetics parameters of its interaction with EspC compared with EspA alone by showing both increased association kinetics (*k*_*a*_ [1/ms]) and increased dissociation kinetics (*k*_*d*_ [1/s]) but similar *K*_*D*_ values (24.8 ± 3 nM); kinetics parameters were significantly different between EspC interacting with EspA/EspD and EspC interacting with EspA alone (Fig. 5C).

**FIG 6:**
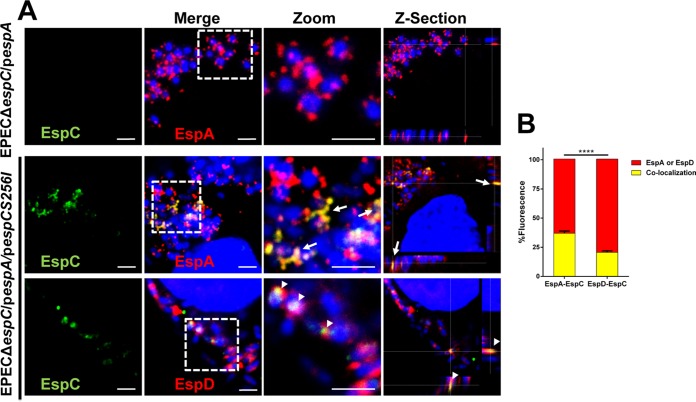
EspC interacts with EspD and EspA filament. (A) Colocalization of EspA-EspC and EspD-EspC. HEp-2 cells were infected with EPEC Δ*espC*(p*espA*) or EPEC Δ*espC*(p*espA*) (*pespCS256I*) in the presence of 0.2% arabinose and 1 mM IPTG for expression of EspC and overexpression of EspA, respectively. Infected cells were fixed but not permeabilized to allow detection of interacting proteins (EspC, EspA, and EspD) outside the cell. Bacteria were immunostained and analyzed by confocal microscopy. EspC was detected with anti-EspC antibodies followed by biotin-SP-conjugated goat anti-mouse IgG and DTAF-conjugated streptavidin, while EspA and EspD were detected with anti-EspA and anti-EspD, respectively, followed by TRITC goat anti-rabbit IgG. DNA was stained with TO-PRO-3 iodine. Maximal projections of the three channels (merged images) and the green channel (EspC) are shown. White squares represent the zoom panels, and middle sections of the three channels were analyzed in *z*-section. Bar, 2 μm. Bacteria and cell nuclei are indicated in blue; depending on the panel label, either EspA or EspD is labeled in red in the last line of panels. Arrows indicate EspA-EspC interaction. Arrowheads indicate EspD-EspC interaction. (B) Percentages of colocalization between either EspA and EspC or EspD and EspC. Nine optical fields (as shown in panel A) from three independent experiments were used to quantify results using red marks (representing either EspA or EspD) and for colocalization of either EspA/EspC or EspD/EspC as yellow marks. Data are means ± SEM of results from three independent experiments. Statistical analysis was performed using two-way ANOVA and Sidak’s test (****, *P* < 0.0001).

In order to better understand the interactions among EspC, EspD, and EspA, we decided to investigate these interactions during EPEC infection using confocal microscopy to detect the interaction of EspC with EspD and EspA filaments protruding from the bacteria and contacting the host cell plasma membrane. To do this, we engineered a construction to overexpress EspA (EPEC Δ*espC*/p*espA*) since it was previously shown that EspA overproduction increases the size of EspA filaments, allowing better detection (36). In addition, we used the *espC*-complemented strain of the latter construction but used the *espCS256I* gene (EPEC Δ*espC*/p*espA*/*pespCS256I*) to avoid the possibility of any proteolytic activity during these interactions. Epithelial cells were infected with these two constructions and analyzed by confocal microscopy using anti-EspC, anti-EspD, and anti-EspA antibodies over nonpermeabilized cells to detect only the EspC that is outside the cells. As expected, cell infection by EPEC Δ*espC*/p*espA* showed blue bacterial signal (bacterial DNA stained with TO-PRO-3 iodide) adhered to the cells and decorated by large red EspA filaments, detected as red dots decorating blue bacteria, but no detection of EspC due to the fact that the construction is mutated in *espC* (Fig. 6A). When the cells were infected with the construction complemented with *espC* (EPEC Δ*espC*/p*espA*/p*espCS256I*), various infected cells were detected with adhered blue bacteria and red EspA filaments colocalizing with secreted EspC (detected as yellow filaments) on the cell surface of nonpermeabilized cells (Fig. 6A). Interestingly, EspA-EspC colocalization occurred along the EspA filaments as detected in *z*-sections of a middle section of infected cells (Fig. 6A; see *z*-section data). Remarkably, when similarly infected cells were immunolabeled with anti-EspC and anti-EspD, infecting blue bacteria were decorated with red EspD marks and some of them colocalized with green EspC marks (Fig. 6A). Thus, EspC/EspD colocalization was prominently detected as punctuated (dotted) marks (Fig. 6; see *z*-section data), but the EspC marks were not localized along the EspD marks such as had occurred between EspC and the EspA marks, suggesting that EspC was interacting with EspD in one extreme of the EspA filaments, probably the tip, where EspD and EspA were interacting. Quantification of nine optical fields (shown in Fig. 6A) from three independent experiments showed that 35% of EspA colocalized with EspC from the total EspA marks (100%), while the level of colocalization between EspD and EspC was significantly lower (20% of the total EspD marks) (Fig. 6B). These data indicate that EspC interacts with EspA filaments and suggest that EspD-EspC interactions occurred in the tip of the filaments, further suggesting that EspC might have been gaining entry to the cell through the EspD/EspB pore in the connection with the EspA filament.

### EspC is getting inside the cells through the EspD/EspB pore.

**FIG 7:**
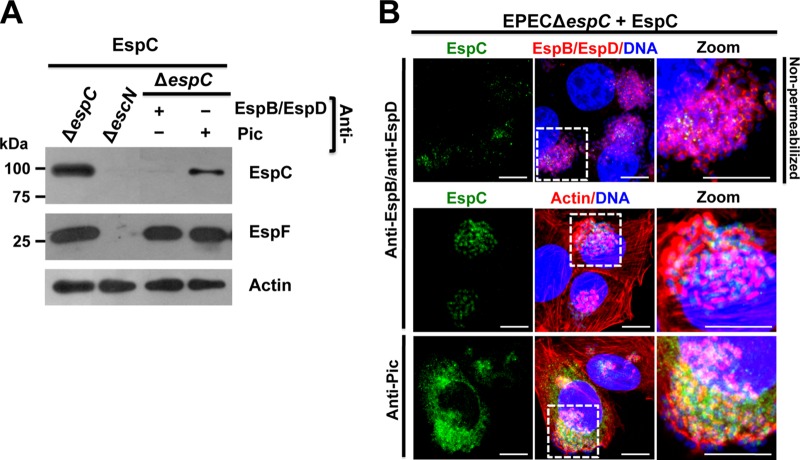
EspC translocation inside epithelial cells is blocked by anti-EspB/anti-EspD antibodies. (A) EspC is not detected in the cytoplasmic fraction by using anti-EspB/anti-EspD antibodies. HEp-2 cells were infected with EPEC Δ*espC* for 1 h and incubated with either anti-EspB/anti-EspD or anti-Pic antibodies for 45 min; finally, exogenous EspC (0.9 μM) was added to the cells and incubated for 2 h. Cytoplasm fractions of infected cells were analyzed by Western blotting using either anti-EspC or anti-EspF antibodies, and actin detection was used as a loading control. An image representative of results of four independent experiments is shown. (B) EspC internalization is inhibited by blocking the EspB/EspD pore. HEp-2 cells were infected and treated with the antibodies and exogenous EspC as described for panel A. In the first panel row, the cells were not permeabilized and were processed for confocal microscopy for decorating the EspB/EspD pores where the bacteria were interacting with the epithelial cells. EspC internalization was detected in cells permeabilized and processed for confocal microscopy. EspC was detected with anti-EspC antibodies followed by anti-mouse antibodies conjugated to fluorescein isothiocyanate (FITC; green). EspB/EspD proteins were detected with anti-EspB and anti-EspD antibodies, respectively, followed by TRITC goat anti-rabbit IgG (red). Actin cytoskeleton was detected with rhodamine-phalloidin (red) and DNA with TO-PRO-3 iodide (blue). Representative images from three independent experiments are shown. The white squares are the zoom panels. Bar, 5 μm.

In order to test the hypothesis that EspC was using the translocon pore inserted into the host cells and connected to the EspA filaments protruding from the bacteria to get inside the cells, we used the EspC exogenous model during cell infection by EPEC Δ*espC* and anti-EspB/anti-EspD antibodies to cause steric hindrance of pore access. Epithelial cells were infected with EPEC Δ*espC*; after bacterial adhesion (1 h), anti-EspB/anti-EspD antibodies were added (45 min), and then exogenous EspC was added and the mixture was incubated for 2 h. After infection, the cells were fractionated and the cytoplasmic fraction was analyzed for EspC translocation by Western blotting. Similar control experiments were performed by using unrelated antibodies such as anti-Pic antibodies (Pic is also an autotransporter protein secreted by enteroaggregative *E. coli*), which were used instead of the anti-EspB/anti-EspD antibodies. In cells infected with EPEC Δ*espC* and exogenous EspC but without any antibody, EspC, as well as a well-known effector protein, EspF, used as a control, was detected in the cytoplasmic fraction, while none of these proteins were detected in cells infected with EPEC Δ*escN* (a T3SS mutant) and exogenous EspC (Fig. 7A). Remarkably, the steric hindrance of pore access by the use of anti-EspB/anti-EspD antibodies during the infection by EPEC Δ*espC* in the presence of purified EspC blocked the exogenous EspC translocation to the cytoplasmic fraction but not the EspF translocation, which was detected in the cytoplasm fraction in an amount similar to that detected at 4 h of infection in an infection kinetics experiment using wild-type EPEC (data not shown). Meanwhile, anti-Pic antibodies were unable to block EspC translocation under the same conditions and both EspC and EspF were detected in the cytoplasmic fraction (Fig. 7A). These data indicate that the steric hindrance mimicked by adding anti-EspB/anti-EspD antibodies after EPEC-epithelial cell contact selectively blocked EspC internalization from the external side but not EspF translocation occurring from the internal side of the EspA filament.

**FIG 8:**
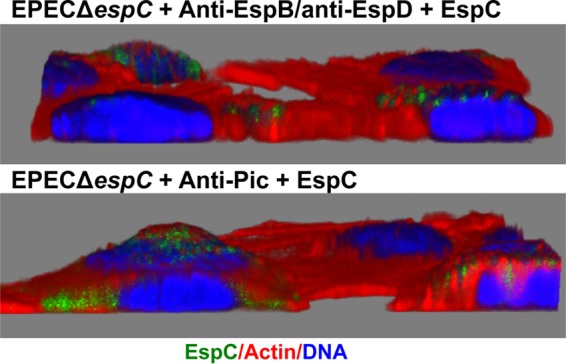
3D reconstruction for detecting the lack of EspC translocation by EspD/EspB pore blockage. HEp-2 cells were infected with EPEC Δ*espC* for 1 h and then incubated with either anti-EspB/anti-EspD or anti-Pic antibodies for 45 min; finally, exogenous EspC (0.9 μM) was added to the cells and incubated for 2 h. EspC translocation was detected in cells permeabilized and processed for confocal microscopy. EspC was detected with anti-EspC antibodies followed by anti-mouse antibodies conjugated to fluorescein isothiocyanate (FITC; green). Actin cytoskeleton was detected with rhodamine-phalloidin (red) and DNA with TO-PRO-3 iodide (blue). Each 3D image is a confocal reconstruction from a total of 50 optical slices at 0.16 μm thickness on the *z* axis.

In order to further support the latter findings, we first sought to label EspD/EspB pores with the anti-EspB/anti-EspD antibodies (used for the steric hindrance experiments) and to detect its colocalization with EspC. Thus, epithelial cells were infected with EPEC Δ*espC*; after bacterial adhesion (1 h), anti-EspB/anti-EspD antibodies were added (45 min), and then exogenous EspC was added and the mixture was incubated for 2 h. These treated cells were then fixed but not permeabilized (to better observe the EspD/EspB pore formation in the plasma membrane), and the anti-EspB/anti-EspD antibodies were immunostained by using a secondary antibody (goat anti-rabbit IgG) labeled with TRITC (tetramethylrhodamine isothiocyanate) and EspC using anti-EspC antibodies followed by a secondary antibody [fluorescein isothiocyanate (FITC) affiniPure *F*(ab′)2 fragment donkey anti-mouse IgG antibody], while the bacterial and cell DNA was detected by the use of TO-PRO-3 iodide. Surprisingly, we were able to detect the EspD/EspB pore (red donuts) on the infected cell surrounding the infecting bacteria (blue). The presence of these decorating marks strongly suggests that the anti-EspB/anti-EspD antibodies were effectively physically blocking the access to the pore. Furthermore, some of these pore protein marks were detected colocalizing with EspC (yellow) outside the cells, as better seen in the zoom panel (Fig. 7B). More excitingly, in cells that had been permeabilized (to observe EspC translocation), it was possible to detect EspC translocation blockage by this steric hindrance using the anti-EspD/anti-EspB antibodies in infected cells by detecting EspC (green), actin cytoskeleton using rhodamine-phalloidin (red), and bacterial and cell DNA using TO-PRO-3 iodide (blue). Thus, EspC green marks were confined to the bacterial microcolony without getting inside the epithelial cells and no colocalization with the actin cytoskeleton was detected. On the other hand, the use of unrelated antibodies (anti-Pic) did not block the EspC translocation inside the cells. EspC green marks were dispersed along the cells, and they were colocalizing with actin fibers in some areas (Fig. 7B). Confocal microscopy using three-dimensional (3D) reconstruction of *z*-sections clearly showed the inhibition of EspC translocation using anti-EspD/anti-EspB antibodies and its internalization when the unrelated antibodies were used (Fig. 8).

## DISCUSSION

EspC protein has to get inside the cells in order to cleave their intracellular targets such as fodrin and focal adhesion proteins such as FAK and paxillin (27) as well as proteins related to the apoptosis cascade such as pro-caspase 3 (28). Thus, a key step for the cell damage induced by EspC is its internalization process. Moreover, EspC translocation inside the epithelial cells appears to involve a singular translocation mechanism because, after EspC secretion by the T5SS and once in the extracellular medium, EspC is efficiently translocated into the epithelial cells in a T3SS-dependent way (32). Here, we have shown how a protein, once secreted to the milieu, is then incorporated into the T3SS to enter the cells through its interaction with the translocator proteins, the EspA filaments, and the EspD/EspB pore. These findings reveal a new translocation mechanism operating through the T3SS which does not involve the passage of the protein inside the injectisome that connects the bacterial and the host cell cytoplasms.

We have previously reported that EspC binds to EspA, but not EspB; however, strains with mutations in either of the genes prevented EspC translocation inside the cells (32). Here we showed that EspC is indeed able to interact with EspA by using sequences encoded in the Middle segment of EspC and (to a lesser extent) the corresponding NH_2_ segment but not the COOH segment. In fact, the Middle segment has a higher affinity for EspA than the full-length EspC protein. Additionally, the equilibrium dissociation constant (*K*_*D*_) in SPR experiments performed using His-EspA was also lower for the Middle segment (3.43 nM) than for the full-length EspC protein (25.11 nM) and was similar to that seen with the EspA-EspA interaction for filament formation, whose *K*_*D*_ was 2.64 nM. Consistently, results of colocalization experiments examined using confocal microscopy showed that EspC is able to decorate EspA filaments. Furthermore, affinity chromatography experiments showed that EspC increased its affinity for EspA in the presence of EspD and SPR analyses showed that EspC increases the association kinetics (*k*_*a*_) and disassociation kinetics (*k*_*d*_) for EspA-EspD in comparison with EspA alone. In fact, by using a completely different approach, it has previously been found that EspC preferentially targets EspA associated with EspD, compared to EspA alone (37). These data, taken together, suggested the possibility that EspC could bind to EspA filaments in order to reach the tip of these filaments where EspA-EspD is interacting and where EspA-EspC interactions may be easily disassociated. In fact, EspC does not interact with EspB, neither EspA with EspB, but EspA strongly interacts with EspD (17), and this interaction increased EspC binding to EspA and also the disassociation kinetics, as shown here. Thus, our findings support the prediction of the topology of the EPEC translocator proteins (EspA binds to EspD but not EspB) in the host membrane previously reported (17).

Interestingly, even though the NH_2_ segment and (mainly) the Middle segment of EspC are able to bind EspA, only the Middle segment was required for EspC translocation; cellular fractionation and Western blotting as well as confocal microscopy analyses showed that the full-length EspC protein and the Middle segment were able to get inside the cell but not the NH_2_ segment or the COOH segment. The requirement of this Middle domain for translocation was confirmed by using an EspC protein deleted in its Middle segment. As with other SPATE proteins, EspC proteins are modular proteins with diverse domains and motifs. Thus, besides its characteristic autotransporter signal sequence and passenger and translocation unit, EspC contains a serine protease motif (TGGDS^256^GS) that is located in the NH_2_ segment (aa 55 to 381) and whose 37-kDa segment maintained its proteolytic activity as demonstrated here (Fig. 3D). Therefore, the sequence for EspC translocation into the Middle segment (aa 382 to 708) is separated from this catalytic active site, constituting a novel identified translocation motif. Interestingly, the precise internalization sequence for EspC is in 21 aa (residues 429 to 449) of the Middle segment. However, this translocation motif is not needed for EspA binding, suggesting that the second loop could be playing the binding role, while the translocation motif is the β-helical stem (38, 39).

Remarkably, the 21-aa translocation sequence of EspC was identified by its homology with the translocation domain of YopH (34, 40) and the inability of EspC protein lacking this sequence to get inside the host cell (Fig. 6 and 7). Thus, EspC translocation needs the T3SS (32) and the translocation motif of EspC (residues 430 to 448). Consistently, it has been published that YopH translocation of externally added YopH requires a functional T3SS and a specific translocation domain in the effector protein (34).

The increasing association and dissociation of EspC found with EspA-EspD in comparison with EspA alone suggest that EspC could be traveling to the EspD/EspB translocator pore; in fact, we have previously shown that EspC translocation does not occur for EPEC mutated in the *espA* or *espB* gene (32). We have shown here and previously that EspC binds to EspA but not to EspB (32); however, the predicted translocator model for EPEC suggests that EspA is interacting with the needle protein EscF and with EspD, EspD is interacting with EspA and EspB, and EspB is interacting only with EspD (17) in a way similar to that which occurs for related *Shigella* translocon components (41). These data suggest that EspC is moved externally on the EspA filaments to the EspD/EspB pore, where its association with EspA is increased and its disassociation kinetics is also increased, causing the release of EspC to reach the EspD/EspB pore, where the translocation motif must play an important role in gaining access inside the cell. Indeed, the use of anti-EspD and anti-EspB antibodies, to cause a steric hindrance of the access of EspC through the pore, effectively blocked the EspC translocation inside the cells as detected by cellular fractionation and confocal microscopy. These data indicated that, in addition to being required for EspA binding and the translocation motifs, the EspB/EspD pore is also required to allow EspC translocation. Similarly, the complementation by extracellular addition of purified YopH effector *in vitro* requires the T3SS translocator proteins (YopB and YopD in *Yersinia*) and the specific translocation domain located in the effector protein (34). Together, these data suggest that the interaction between the translocators and the translocation domain in the effector (YopH or EspC) is probably necessary for translocation of the effector across the plasma membrane of the target cell. According to the results of our experiments using SPR and those for blocking the translocation pore, EspA filaments must be connected to the EspD/EspB pore as a complex in order to allow EspC translocation. In fact, we previously found that bacterium-host cell contact is required for EspC translocation (29). Moreover, we have also shown that the cell damage induced by other EPEC effectors (such as those inducing cell membrane damage) does not play a role in EspC translocation, since this translocation does not occur when cells are infected with EPEC Δ*espC* for 1, 2, or 3 h (to allow the injection of translocator and effector proteins into or onto the host cells) and then these bacteria are eliminated with tetracycline followed by incubation of these epithelial cells with exogenous EspC. This EspC translocation occurs only during the infection process (32).

Excitingly, our findings and those reported for YopH translocation indicate that this novel mechanism of translocation of extracellular effectors by the T3SS is conserved among different T3SS-dependent pathogens. Thus, EspC can be translocated when it is expressed in EHEC and rabbit EPEC (REPEC) or by adding the purified EspC protein; these two A/E pathogens lack the *espC* gene but contain a functional T3SS (32). Furthermore, purified YopH coated on *S. enterica* serovar Typhimurium strains was translocated by the bacteria in a SPI-1-dependent manner, which suggests that T3SS-dependent translocation is required (34).

Thus, this newly identified translocation mechanism mediated by the T3SS, in which extracellular (secreted) effectors can be translocated by a T3SS-dependent mechanism, involves the binding of the protein to the bacterial surface (YopH) or the EspA filament (EspC), a translocation motif, and the translocator pore by which the protein gets access into the host cell. Interestingly, this novel mechanism does not exclude the microinjection model; therefore, T3SS can be functioning as a conduit or a railway. These two mechanism operate in parallel because it is possible to block EspC translocation by using antibodies against EspD and EspB for blocking (through external steric hindrance) the access to the pore; however, EspF is still efficiently translocated into these host cells, a process that is believed to occur in one step from the bacterial cytosol to the target-cell cytoplasm through a conduit created by the T3SS.

## MATERIALS AND METHODS

### Bacterial strains.

Characteristics of the strains used in this study are listed in Table 1. All strains were routinely grown in Luria-Bertani (LB) broth aerobically at 37°C. When necessary, the medium was supplemented with ampicillin (100 µg/ml), kanamycin (50 µg/ml), or chloramphenicol (35 μg/ml).

**TABLE 1:** *Escherichia coli* strains, plasmids, and targets used in the present study

Strain, plasmid, or target	Relevant characteristic(s) or sequence	Reference or source
*E. coli* strains		
HB101 (pJLM174)	Minimal *espC* clone, cloned in pBAD30	23
HB101 (p*espCS256I*)	*espC* clone mutated in residue S256	26
Δ*espC*	EPEC E2348/69 Δ*espC*, EspC isogenic mutant	24
Δ*escN*	EPEC E2348/69 Δ*escN* T3SS mutant (CVD452)	44
BL21(D3)pLysS	Expression strain	Invitrogen
BL21 (p*espA*)	Minimal *espA* clone (pMSD2), cloned in pET28a	14
Δ*espC*(p*espA*)	EPEC E2348/69 Δ*espC* isogenic mutant transformed with pMSD2	This study
Δ*espC*(p*espA*) (*pespCS256I*)	EPEC E2348/69 Δ*espC* isogenic mutant transformed with pMSD2 and p*espCS256I*	This study
BL21(p*espA*) (p*espD*)	BL21 transformed with pMSD2 and *pMEespD*	This study
Plasmids		
pJLM174	4-kb fragment of *espC* gene cloned into pBAD30	23
p*espCS256I*	pJLM174 with a site directed mutagenesis of Ser to Ile	26
p*espD*	1-kb fragment of *espD* gene cloned into pET19b (pME*espD*)	B. González-Pedrajo
p*espA*	0.576 kb fragment of *espA* gene cloned in pET28a	W. P. Elias
pRSET-A/NH_2_, Middle or COOH	0.97-kb fragments of *espC* gene cloned into pRSET-A	This study
pRSET-A/*espC*ΔMiddle	1.9-kb fragment of *espC* gene cloned into pRSET-A	This study
pRSET-A/*espC*	2.9-kb fragment of *espC* gene cloned into pRSET-A	This study
pRSET-A/*espC*Δ430-448	2.8 kb fragment of *espC* gene cloned into pRSET-A	This study
Targets		
NH_2_	F: 5′ GCTCAACTAAATATTGATAATGT 3′; R: 5′ TTTATTATTACCAGTGACAGTAT 3′	This study
Middle	F: 5′ ACATTCAAGGGTGCCGGG 3′; R: 5′ TCCAAAATAACTGCTGCTCTCA 3′	This study
COOH	F: 5′ ATTATTGACCTCTGTCAGGAA 3′; R: 5′ AATGTTACGCTGAATAATCACTC 3′	This study
ΔMiddle	F: 5′ ACGCTGAATAATCACTCATTACTG 3′; R: 5′ TGTTTTATTATTACCAGTGACAGT 3′	This study
EspC	F: 5′ GCTCAACTAAATATTGATAATGT 3′; R: 5′ AATGTTACGCTGAATAATCACTC 3′	This study
EspCΔ430-448	F: 5′ CATGGTGTTGTAAAAATTAATCATAGTGCAGCG 3′; R: 5′ ATCCCCCGTTTTAAGATTATTACC 3′	This study

### Molecular cloning and constructions for recombinant proteins.

For cloning of EspC segments (NH_2_, Middle, and COOH; 978 bp) and EspC, the sequences were amplified by PCR using pJLM174 containing the *espC* gene as the template and the PCR products were cloned in pRSET-A vector. Plasmids pRSET-A/*esp*CΔMiddle and pRSET-A/*esp*CΔ430-448 were derived from pJLM174 using an inverse PCR to exclude the desirable sequences. The oligonucleotide sequences used are listed in Table 1.

### Protein expression and purification.

To purify EspC and EspCS256I, HB101(pJLM174) and HB101(p*espCS256I*) strains were grown overnight in LB broth supplemented with ampicillin plus 0.2% arabinose with shaking. Supernatants were obtained by centrifugation at 3,124 × *g* for 20 min, filter sterilized, and concentrated 100-fold by filtration in 100-kDa-cutoff Ultrafree centrifugal filter devices (Millipore, Bedford, MA). The concentrates were filter sterilized again, and proteins were quantified by the micro-Bradford method (42).

Expression and purification of recombinant 6×His tag proteins were performed as described previously (38) with a few modifications. Bacterial pellets from 100-ml cultures were sonicated in 10 ml of lysis buffer (50 mM NaH_2_PO_4_, 300 mM NaCl, 5 mM imidazole, pH 8.0) supplemented with 10 mg of Sarkosyl NL and 10 µl of Triton X-100 and were then centrifuged at 9,000 × *g*. The pellets were sonicated in 1 ml of lysis buffer supplemented with 10 mg of Sarkosyl NL until the pellets were clarified. Samples were centrifuged at 20,598 × *g*, and the supernatants were diluted in lysis buffer. Each protein solution was purified by using Ni-NTA agarose columns (Qiagen, Hilden, Germany), and elution products were dialyzed against phosphate-buffered saline (PBS) and analyzed by SDS-PAGE.

### Overlay assays.

Blot overlay assays were performed as previously reported (43) with a few modifications. Purified proteins (EspC, His-NH_2_, His-Middle, His-COOH, His-EspCΔMiddle, and His-EspA) (1 µg) were separated by SDS-PAGE and transferred to PVDF membranes. EspC and derivate proteins (His-NH_2_, His-Middle, His-COOH, His-EspCΔMiddle, His-EspC, and His-EspCΔ430-448) (2.5, 5, 10, and 20 nM) were adsorbed to nitrocellulose membranes for dot blot overlay using a Bio-Dot apparatus (Bio-Rad, Hercules, CA). EspC, His-NH_2_, His-EspCΔMiddle, His-EspC, and His-EspCΔ430-448 were preincubated with 2 mM phenylmethylsulfonyl fluoride (PMSF) before being adsorbed to avoid proteolytic activity. Membranes from the blot and dot blot assays were blocked overnight, incubated with His-EspA at 5 µg/ml in binding buffer (20 mM Tris-HCl, 150 mM NaCl, 0.1% Tween 20, 2 mM CaCl_2_, 5% BSA) for 1.5 h, incubated with mouse anti-EspA antibody for 1 h and then with goat anti-mouse IgG secondary antibody, and (horseradish peroxidase [HRP]) (JIR, West Grove, PA) conjugated for 1 h. The reaction was detected using an Immobilon Western chemiluminescent HRP substrate kit (Millipore, Darmstadt, Germany).

### Surface plasmon resonance.

Kinetics analyses were done in a Reichert (Depew, NY) SR7500DC dual-channel SPR instrument. For analysis of the kinetics of EspA-EspA and EspA-EspC Middle fragment interactions, planar polyethylene glycol/carboxyl sensor slides were used. The flow rate for all steps was determined with phosphate-buffered saline with Tween 20 (PBST) at 50 µl/min. His-EspA was coupled to the sensor by passing N-ethyl-N′-(3-dimethylaminopropyl) carbodiimide–N-hydroxy-succinamide (EDC-NHS) for 5 min, immobilizing His-EspA in 10 mM sodium acetate buffer (pH 4.0) at 20 µg/ml just over the left channel for 8 min, and blocking with 1 M ethanolamine (ETN) for 8 min. For His-EspC-His-EspA and His-EspC-His-EspA/His-EspD kinetics assays, a nickel-nitrilotriacetic acid sensor slide was used. His-EspC was coupled by the use of a His tag only over the left channel of the sensor by passing 40 mM nickel sulfate for 3 min, injecting EDC-NHS for 7 min, immobilizing His-EspC at 60 µg/ml in PBST for 15 min, blocking with ETN for 5 min, and removing non-covalently bound protein with 350 mM EDTA for 3 min. Kinetics analyses were done by injecting five concentrations of ligand (25 to 600 nM) into PBST, and 20 mM NaOH was injected to regenerate the surface for 8 min. Data were recorded using Integrated SPRAutolink software, and BioLogic scrubber 2 software (Campbell, Australia) was used for curve fitting and data analysis. Statistical analyses (unpaired, two-tailed *t* test) were performed with GraphPad Prism 6.0 (San Diego, CA, USA) using the values from 40 different injections. *P* values of <0.05 were considered statistically significant. The data were expressed as means ± standard errors of the means.

### Degradation assays.

Glutathione *S*-transferase (GST)-fodrin was prepared as described previously (26). Purified GST-fodrin (2.5 µg) was incubated with EspC His-NH_2_ (2 µg) for different times (0.5 to 4 h). All the reactions were stopped with Laemmli buffer, and the reaction products were analyzed by SDS-PAGE. For inhibition experiments, His-NH_2_ was preincubated with 2 mM PMSF at 37°C for 30 min.

### Exogenous EspC translocation assays.

HEp-2 cells were seeded in 35-mm-diameter culture dishes to reach 100% confluence. The cells were infected using a model of exogenous protein application during the infection, in which activated EPEC isogenic mutants were simultaneously incubated in Dulbecco’s modified Eagle’s medium (DMEM) with 1% d-mannose at a multiplicity of infection (MOI) of 10 during 4 h and with EspC, His-NH_2_, His-Middle, His-COOH, His-EspCΔMiddle, His-EspC, or His-EspCΔ430-448 (0.9 μM). After treatment, the cells were fractionated as previously reported (32). Protein translocation was analyzed in the cytoplasmic fraction by Western blotting using mouse anti-EspC or mouse anti-actin antibodies (loading control) and a secondary antibody goat anti-mouse IgG (HRP) conjugation (Thermo Fisher, Waltham, MA). The reactions were detected using an Immobilon Western chemiluminescent HRP substrate kit (Millipore, Darmstadt, Germany). For immunofluorescence assay, HEp-2 cells were cultivated in 8-well Lab-Tek chamber slides (Nalgene Nunc International, USA) and were infected for 3 h using the protein exogenous model as mentioned above. After cell treatment, the coverslips were fixed and immunostained with mouse anti-EspC antibodies, followed by a fluorescein isothiocyanate (FITC) affiniPure *F*(ab′)2 fragment donkey anti-mouse IgG antibody (JIR, West Grove, PA). Actin cytoskeleton was stained with phalloidin-TRITC (tetramethylrhodamine isothiocyanate) and the nucleic acids with TO-PRO-3 iodide (Life Technologies, Inc., USA). The slides were mounted and visualized with a Leica TCS SP8 confocal tandem microscope (Leica, Wetzlar, Germany).

### Immunofluorescence assay for visualization of EspA filaments.

Overnight cultures of EPEC Δ*espC*/p*espA* or EPEC Δ*espC*/p*espA*/p*espCS256I* were grown with 0.2% arabinose for 1 h before the activation. HEp-2 cells were infected with EPEC Δ*espC*/p*espA* or EPEC Δ*espC*/p*espA*/p*espCS256I*. HEp-2 cells were infected for 1 h, and fresh DMEM with 1 mM IPTG (isopropyl-β-d-thiogalactopyranoside) was added for 2 h more. Following infection, the cells were fixed and inmunostained using rabbit anti-EspD or rabbit anti-EspA and mouse anti-EspC or mouse anti-EspC and rabbit anti-EspD antibody. Between the application of one primary antibody and the next one, a step of blocking with 1% BSA was added. After incubation with the primary antibodies, the following secondary antibodies were used: TRITC affiniPure goat anti-rabbit IgG and biotin-SP-conjugated goat anti-mouse IgG or biotin-SP-conjugated goat anti-mouse IgG and TRITC affiniPure goat anti-rabbit IgG antibodies, respectively. The biotin antibodies were detected with dichlorotriazinylamino fluorescein (DTAF)-conjugated streptavidin or tetramethylrhodamine isothiocyanate (TRITC)-conjugated streptavidin (JIR, West Grove, PA). Preparations were stained with TO-PRO-3 iodide for nucleic acids.

### Complex isolation assay.

The purified proteins His-EspA and His-EspA/His-EspD (60 µg) were incubated with EspCS256I (20 µg) for 2 h at 4°C. After that, the interactions were captured by the use of Ni-NTA agarose, and the complexes were eluted, separated by SDS-PAGE, and analyzed by Western blotting with anti-EspC, anti-EspD, and anti-EspA antibodies and goat anti-mouse IgG (HRP)-conjugated antibody or goat anti-rabbit IgG (HRP)-conjugated antibody (Thermo Fisher, Waltham, MA) as the secondary antibody. The reaction was detected using an Immobilon Western chemiluminescent HRP substrate kit (Millipore, Darmstadt, Germany).

### Translocon blocking assays.

HEp-2 cells, grown in 48-well tissue culture dishes until 90% confluence was reached, were infected with activated cultures of the EPEC isogenic mutant for 1 h. The cells were then incubated with anti-EspB and anti-EspD or anti-Pic antibodies (an irrelevant antibody that recognizes another autotransporter protein) for 45 min. The monolayers were washed, and purified EspC was added during 2 h. The cytoplasmic fractions were analyzed by Western blotting. The confocal microscopy assays were done as described above with a few modifications. To visualize the blocking antibodies (anti-EspB and anti-EspD), the fixed cells were incubated with (TRITC) goat anti-rabbit antibody without permeabilization. To observe EspC translocation, HEp-2 cells were permeabilized and inmunostained as described above.
